# PRI: Re-Analysis of a Public Mass Cytometry Dataset Reveals Patterns of Effective Tumor Treatments

**DOI:** 10.3389/fimmu.2022.849329

**Published:** 2022-05-03

**Authors:** Yen Hoang, Stefanie Gryzik, Ines Hoppe, Alexander Rybak, Martin Schädlich, Isabelle Kadner, Dirk Walther, Julio Vera, Andreas Radbruch, Detlef Groth, Sabine Baumgart, Ria Baumgrass

**Affiliations:** ^1^ German Rheumatism Research Center (DRFZ), A Leibniz Institute, Berlin, Germany; ^2^ Bioinformatics, Institute of Biochemistry and Biology, University of Potsdam, Potsdam, Germany; ^3^ Bioinformatics, Max Planck Institute of Molecular Plant Physiology, Potsdam, Germany; ^4^ Laboratory of Systems Tumor Immunology, Friedrich-Alexander University of Erlangen-Nürnberg (FAU) and Universitätsklinikum Erlangen, Erlangen, Germany; ^5^ Department of Rheumatology and Clinical Immunology, Charité, Campus Berlin Mitte, Berlin, Germany; ^6^ Institute of Immunology, Core Facility Cytometry, University Hospital Jena, Jena, Germany

**Keywords:** multi-parametric analysis, re-analysis, combinatorial protein expression, high-dimensional cytometry data, mass cytometry data, pattern perception

## Abstract

Recently, mass cytometry has enabled quantification of up to 50 parameters for millions of cells per sample. It remains a challenge to analyze such high-dimensional data to exploit the richness of the inherent information, even though many valuable new analysis tools have already been developed. We propose a novel algorithm “pattern recognition of immune cells (PRI)” to tackle these high-dimensional protein combinations in the data. PRI is a tool for the analysis and visualization of cytometry data based on a three or more-parametric binning approach, feature engineering of bin properties of multivariate cell data, and a pseudo-multiparametric visualization. Using a publicly available mass cytometry dataset, we proved that reproducible feature engineering and intuitive understanding of the generated bin plots are helpful hallmarks for re-analysis with PRI. In the CD4^+^T cell population analyzed, PRI revealed two bin-plot patterns (CD90/CD44/CD86 and CD90/CD44/CD27) and 20 bin plot features for threshold-independent classification of mice concerning ineffective and effective tumor treatment. In addition, PRI mapped cell subsets regarding co-expression of the proliferation marker Ki67 with two major transcription factors and further delineated a specific Th1 cell subset. All these results demonstrate the added insights that can be obtained using the non-cluster-based tool PRI for re-analyses of high-dimensional cytometric data.

## Introduction

In the last decade, cytometric technologies have tremendously progressed, allowing for parallel measurement of up to 20 proteins with flow cytometry and 50 proteins with mass cytometry (1). This progress shifted the analysis strategy from conventional consecutive manual gating to multidimensional data processing (2, 3). To cope with the emerging complexity of multiparametric single-cell data, approaches such as nonlinear dimensionality reduction [such as UMAP (4) and vi-SNE (5)] and various clustering methods [such as PhenoGraph (6), Citrus (7), and FlowSOM (8)] have been implemented to analyze, visualize, and interpret high-dimensional cytometric data [reviewed in (9–11)]. All these tools have different advantages and disadvantages (12), their main obstacles usually lying in lack of both interpretability and reproducibility, complexity of the algorithms, and comparability between samples and groups (11).

To partially address this bottleneck, we developed a reproducible, semi-automated method for analyzing and visualizing cytometric data, which we called “pattern recognition of immune cells” (PRI). This algorithm relies on a binning approach that, on the one hand, leads to feature engineering based on the combinatorics of three protein markers. On the other hand, the approach produces bin plots that represent a semi-continuous visualization of marker intensities and can reveal characteristic patterns. We successfully used this new approach to identify a super-functional Tfh-like cell subset from our own flow cytometry data (13).

Here, we investigated whether the bin-based PRI approach is suitable for re-analysis of mass cytometry datasets and whether it can generate new immunological insights. To this aim, we selected a publicly available dataset from Spitzer et al. who identified a CD4^+^ T-cell subpopulation that discriminates between ineffective and effective treatment of mouse carcinoma (14). Using pseudo-multiparametric visualization and a four-parametric approach with PRI we could further constrain this Th1 population. Furthermore, we used PRI analysis for feature engineering of novel bin parameters for prognosis of effective and ineffective therapy.

## Method

### Datasets

**Figure 1:**
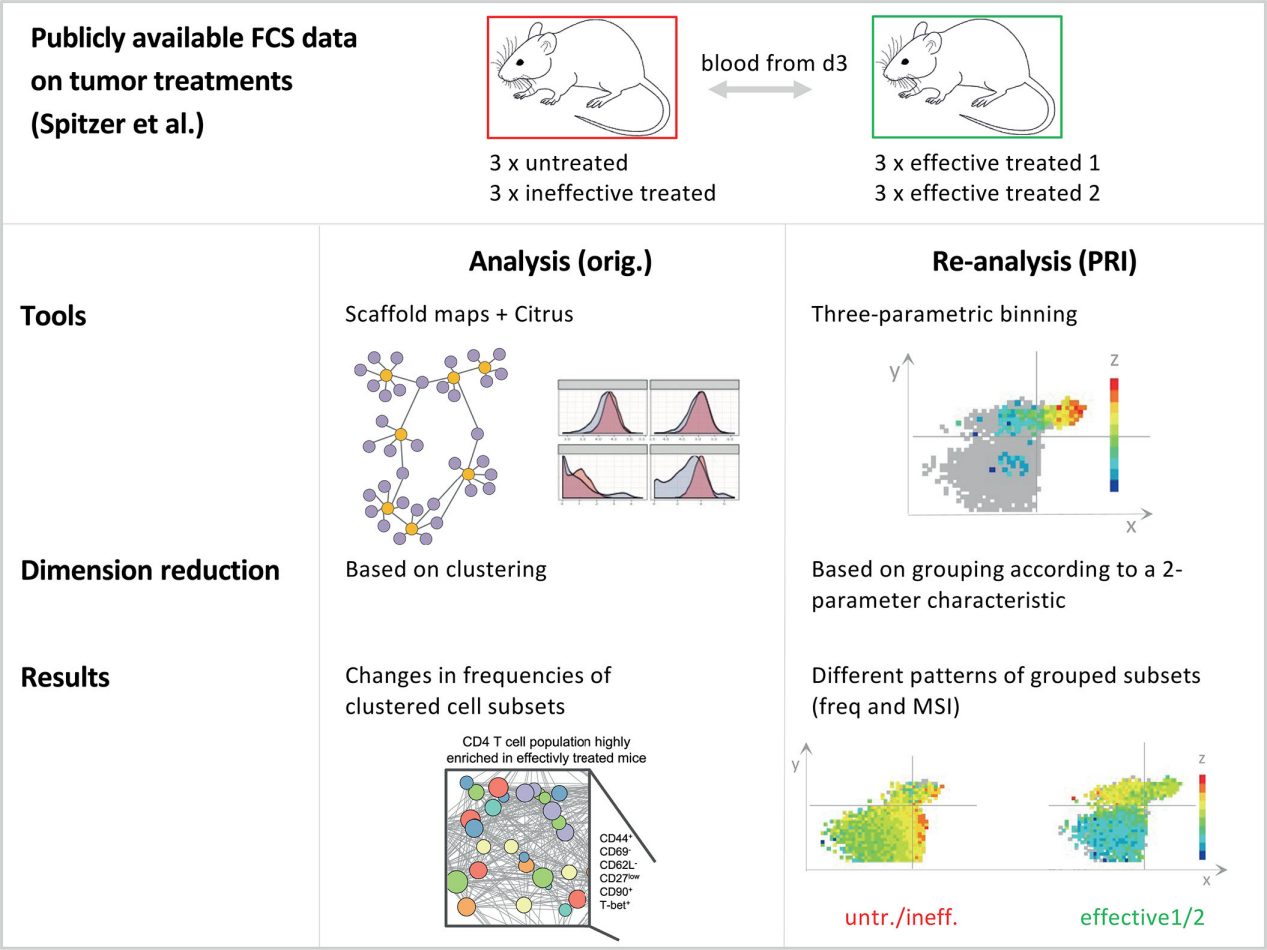
Schematic comparison of the analysis approach with the re-analysis approach. Comparison of the analysis approach of Spitzer et al. (14) and the re-analysis of the data with PRI. Both studies analysed the same publicly available datasets on tumor treatments, while our re-analysis was limited to blood samples from day three from mice. For this dataset, there were a total of four different treatments, which can be divided into the two groups effective and ineffective. Within the ineffective treatment group there were animals that received no treatment (untreated - untr.) and those that received an ineffective (ineff.; anti-PD-1) treatment. In addition, there were effective treatment1 (effective 1; IFN-γ + anti-CD40 + CD1-allo-IgG) and effective treatment2 (effective 2; IFN-γ + anti-CD40 + B6-allo-IgG). For details on the experimental setting see Spitzer et al. The original analysis used common tools in flow cytometry, such as scaffold maps and citrus as well as clustering to reduce dimensions. As a result, the authors found changes in the frequencies of clustered cell subsets. In our bin-based re-analysis (PRI approach) we found different patterns and properties of grouped cell subsets concerning frequencies and MSI (mean signal intensity).

The re-analysis was performed on a dataset from the Cytobank project ‘Spitzer-et-al_Cell_2017’ (https://community.cytobank.org/cytobank/experiments#project-id=1025) obtained from the Cytobank repository (15). It was originally collected by Spitzer et al. (14), who used a common mouse carcinoma model to study four different treatments, of which two were effective and two were ineffective (**Figure 1**). Briefly, the mice were implanted with tumor cells, which were left to grow until they reached a specific size. Then, the mice were shared between four treatment groups. Disease progression and treatment effect were monitored by blood and tissue sampling at different time points. Of these data, we re-analyzed blood samples taken from 12 mice on day three of treatment with 41 biological markers measured for each sample. Thus, a total of six animals were in the effective treatment and originally six animals were in the ineffective treatment. Each treatment group was assigned three animals: untreated (untr.), ineffective treatment (ineff.; anti-PD-1), effective treatment1 (effective 1; IFN-γ + anti-CD40 + CD1-allo-IgG) and effective treatment2 (effective 2; IFN-γ + anti-CD40 + B6-allo-IgG). However, one blood sample from the ineffective treatment (untr. blood3) was excluded from our analysis because of quality reasons (please see below).

### PRI-Analysis Workflow

**Figure 2:**
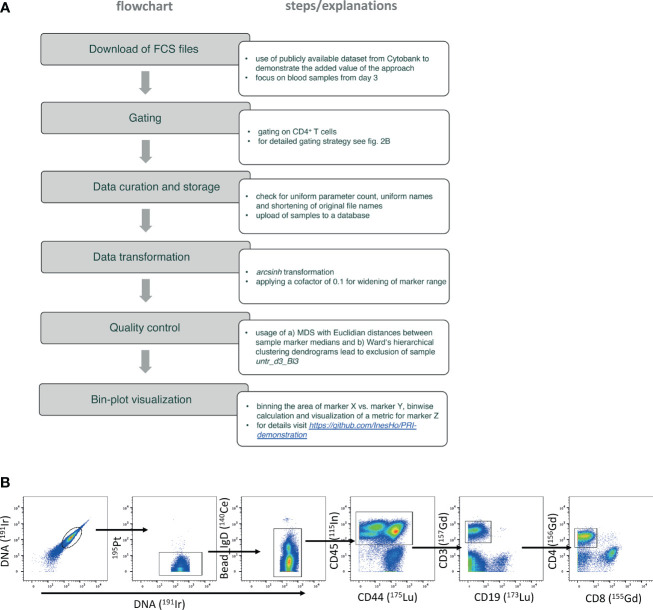
Flowchart of the used PRI-analysis workflow. **(A)** The flowchart lists the step by step details. **(B)** Gating strategy focusing on selecting CD4 T helper cells – plots from left to the right show the identification of live single cells by using the signal of the iridium-DNA-intercalator and the negative expression of cisplatin represented by 195Pt. Left-over internal standard beads were excluded by using high 140Ce signal. CD45 as unique pan-leukocytic marker, and CD3 were used to detect T cells. The very right plot shows the discrimination of CD4 T helper cells from CD8 cytotoxic T cells.

In order to use PRI to visualize and analyze the dataset, some necessary steps to prepare the data must be performed. In **Figure 2A** we briefly summarize all steps that were part of the workflow to achieve the results as we present them here. The following paragraphs describe these steps in more detail.

### Preprocessing of Data and Gating

Precleaned debarcoded data from blood samples were obtained from the public dataset (14). The blood samples were further processed in FlowJo™ v9.9.6 software as is exemplarily shown in the gating strategy in **Figure 2B**.

Here, we gated first on live single cells (191Ir193Ir^+^ and 195Pt- cells) and removed internal standard beads added before CyTOF measurement by using unique 140Ce^++^ signal. Total leukocytes were gated based on CD45^+^ expression. T cells were further selected by CD3^+^CD19- expression. CD4 T helper cells used for the re-analysis in this study were determined by their CD4^+^CD8- expression.

### Quality Control

The quality of the datasets was assessed with conventional metric multidimensional scaling (MDS, see **Supplemental Figure 3A**) performed using the Bioconductor package ‘limma’ (16). Pairwise Euclidean distances of the median expression values for the 41 biological markers in all samples were calculated. It was evident that one blood sample from the untreated group differed in its SI medians from the other samples in this group. A similar behavior of this sample was seen using Ward’s hierarchical clustering (17) from the ‘stats’ package that is part of R (**Supplemental Figure 3B**). Both results and a very low cell count prompted us to exclude sample “untr. blood3” from further examination with PRI.

### PRI Analysis and Bin Plot Visualization

The extracted CD4^+^ T cell populations are imported as FCS files in PRI, where the SI are transformed with inverse hyperbolic sine (arcsinh), commonly used for flow cytometric analysis (18), with a cofactor of 0.1.

The signal intensities of two markers were divided into bins of width 0.2 x 0.2 asinh along the x and y axis for bin plot visualization. For all cells in each bin, different statistical values were calculated and displayed in a color-coded manner as heat maps. Low values are represented as shades of blue, median values as yellow, and high values as red color. The patterns of mean signal intensity of parameter z^+^ cells (MSI^+^) and the mean signal intensity of parameter z for all cells per bin (MSI) are analyzed. Additional statistics are displayed in black and red percentage numbers in each quadrant. Percentages in black indicate the frequency of cells relative to total cells. The frequency of z+ cells is given in red, and is relative to the cells inside the respective quadrants. All events (cells) are included in the analysis but only bins containing five or more cells are displayed, balancing the impact of identifying comparatively rare subpopulations while retaining statistical robustness.

In this publication we present the core functionality of PRI (for details visit https://github.com/InesHo/PRI-demonstration; also available as R-package within the repository). We used the R package ‘flowCore’ (19) to read the FCS files and R-core (20) functionalities to create the plots. The plots for MDS and Ward’s hierarchical clustering presented in the **Supplementary Material** have been created using the R-packages ‘ggplot2’ (21) and ‘dendextend’ (22).

### Quantification and Statistical Analysis

Two-group comparisons (n = 5 vs. n = 6) were statistically assessed by non-parametric Mann-Whitney U test using GraphPad Prism8 Software. Median values were used. P < 0.05 was considered significant, with the numbers of asterisks indicating: *p ≤ 0.05; **p ≤ 0.01; ***p ≤ 0.001; ****p ≤ 0.0001.

## Results

### Basic Concept of the PRI Approach

To evaluate the added value of the PRI approach (13) used in combination with other strategies for the analysis of high-dimensional mass cytometric data, we selected a publicly available dataset (14). Spitzer et al. studied the effectiveness of different immunotherapies in a mouse model of breast carcinoma. From the comprehensive data, we selected the three-day blood dataset, including six mice, from which the authors had described a mixture of CD4^+^ T cell clusters expanded by two different anti-tumor therapies, as revealed by a combination of Scaffold Maps (6) and Citrus analysis (7) (**Figure 1**).

We attempted to use our bin-based approach PRI to further analyze, constrain and visualize the specificities of the described cluster mixture (CD44^+^ CD69^-^ CD62L^-^ CD27low CD90^+^ Tbet^+^). PRI uses an x-y plane divided into equally sized bins and color-codes the intensity or frequency of a z parameter. In this process, PRI semi-continuously analyzes and visualizes the combinatorics of mainly 3 parameters in so-called bin plots. Each axis is informative and intuitively understandable. PRI operates reproducibly and provides combinatorial features such as bin intensity ranges or maximum bin intensity values that are threshold-independent. In cases where frequencies are desired either in bins, quadrants, or cells, it is necessary to manually define thresholds for parameters (e.g., between negative and positive or between low and high values). All these features are useful to compare and classify samples.

**Figure 2** summarizes and describes the work-flow (**Figure 2A**), including the gating strategy (**Figure 2B**). To ensure the quality of the analysis, we excluded one sample from the untreated group (see details in the Methods part).

### Identification of Discriminating Bin Patterns Based on the Maximum PRI Bin Intensities

**Figure 3:**
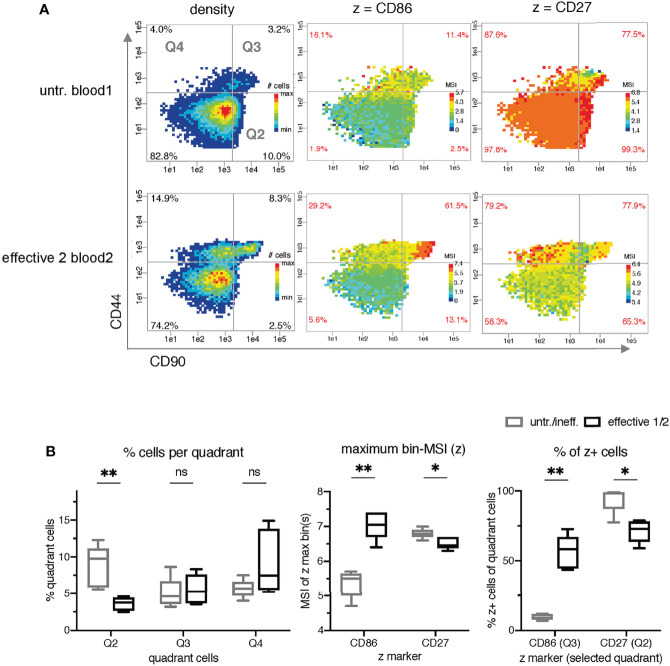
Identification of intensity bin-patterns and bin-values for the classification of mice receiving treatments with differential effects. **(A)** Comparison of the density and mean signal intensity (MSI)-bin patterns of two exemplary samples (untreated blood1 and effective 2 blood2) using semi-continuous binning with CD90 (x-axis) and CD44 (y-axis) and dynamic intensity ranges for z. **(B)** Statistical comparison of three different metrics as z parameter in PRI per sample: the frequency of the quadrant cells (left graph; black quadrant numbers of density plots), the maximal bin-MSI values of CD86 and CD27 (middle graph; max-bin values of the color-coded legend of MSI bin plots), and the frequencies of z^+^ cells per quadrants (right graph; red quadrant numbers of MSI-bin plots of all 11 samples). The ineffective treatment group with untreated (untr.) and ineffectively treated (ineff.) samples is shown in gray and the effective treatment group with effective treatment 1 and 2 in black. P < 0.05 was considered significant, with the numbers of asterisks indicating: *p ≤ 0.05; **p ≤ 0.01; ns stands for non-significant.

**Figure 4:**
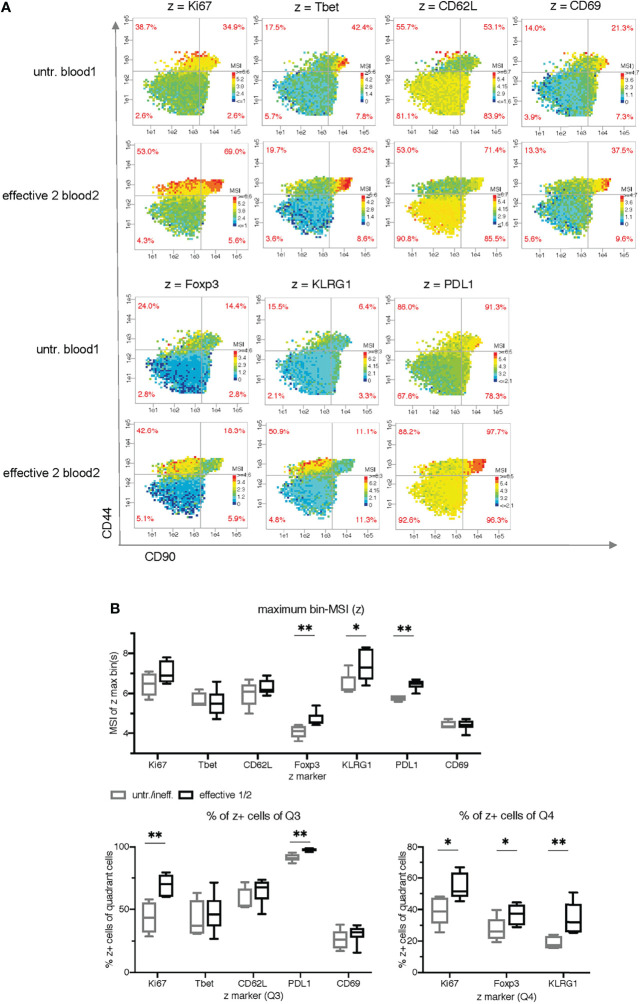
Cell subset characterization using side by side plots and common ranges for each z-parameter. **(A)** Comparison of mean signal intensity (MSI)-bin patterns of two exemplary samples (untreated blood1 and effective 2 blood2) for seven z parameters using common intensity ranges for z (min-max of both samples). **(B)** Statistical comparison of the maximum bin-MSI values per sample (PRI-analysis) and the frequencies of the quadrant cells in Q3 and Q4 (red quadrant numbers of the MSI-bin plots) of all 11 samples for seven z parameters. The ineffective treatment group with untreated (untr.) and ineffectively treated (ineff.) samples is shown in gray and the effective treatment group with effective treatment 1 and 2 in black. P < 0.05 was considered significant, with the numbers of asterisks indicating: *p ≤ 0.05; **p ≤ 0.01.

Using the clustering information of Spitzer et al. together with background immunological knowledge and manual inspection of many 3-parametric overview plots, we decided to use CD90 and CD44 to define the x-y parameter plane. Evidence for CD90 and CD44 as possible x and y markers also comes from the non-redundancy score (NRS) (**Supplementary Table 1**) (23). The selection criteria included a broad intensity range and sufficient grouping of Ki67^high^ cells, as well as Tbet cells. The first bin analysis statistic of the maximum bin MSI values of selected T cell parameters confirmed this choice and revealed statistically significant differences between the two classification groups (5 mice with untreated/ineffective treatment and 6 mice with effective treatments 1/2) for the five z markers CD86, CD27, Foxp3, KLRG1, and PDL1 (**Figures 3B**, **4B**).

Furthermore, the manual inspection of the MSI bin plots with these z parameters revealed different patterns in the case of CD86 and CD27 (**Figure 3A**). For the visualization of these patterns, we used dynamic MSI bin plots, meaning that the z parameter of each is color-coded relatively to its own individual minimum-maximum range of MSI (**Supplemental Figures 1**, **2**). Thresholds were defined for x and y to discriminate CD44^-^ and CD44^+^ cells, as well as CD90^-^/low and CD90high cells.

It is obvious that there are higher CD86 MSI-bins in effective treatment (bin-range right in each plot). In addition, the biggest differences between untreated/ineffective treatment and effective treatments are mainly found in quadrant 3 (Q3). The highest CD27 bins are found in Q2 only in the samples from the untreated/ineffective treatment groups with one exception and the differences in the patterns are not as evident as in CD86.

However, lower cell frequencies in Q2 are also statistically significant in the effective treatment group (**Figure 3B** left). Setting manual thresholds for the z parameters based on histograms similar to those in FlowJo allowed the determination of the frequency of CD86^+^ and CD27^+^ cells in the bin plots (red frequencies in the corners of each quadrant). High frequencies of CD86^+^ in Q3 and low frequencies of CD27^+^ in Q2 were found to be significant for the classification of the samples.

### Cell Subset Characterization by Pseudo-Multiparametric Visualization

To simulate multiparametric visualization and characterize the therapy-expanded cell subsets grouped in Q3 and Q4, we plotted seven additional z markers side by side on the same x-y plane as in **Figure 3** (**Figure 4A**). However, in contrast to **Figure 3A**, we took both exemplary samples (untr. blood1 and effective2 blood2 (IFN-γ + anti-CD40 + B6-allo IgG)) for each z marker and determined a common lower and upper bin-MSI value from both samples together. This provides a direct visualization of differences in intensity of both samples. The PRI feature statistics of all 11 samples (**Figure 4B**) reveals some classification value for Ki67 (frequencies in Q3 and Q4), KLRG1 (maximum bin MSI and frequencies in Q4), Foxp3 (maximum bin MSI and frequencies in Q3), and PDL1 (maximum bin MSI and frequencies in Q3). Interestingly, Tbet shows a tendency of different median values in the frequencies of Q3, although it was a major characteristic of the expanded cell cluster mix in the original work of Spitzer et al.

### Cell Subset Characterization by Four-Parameter Combinatorial Analysis

**Figure 5:**
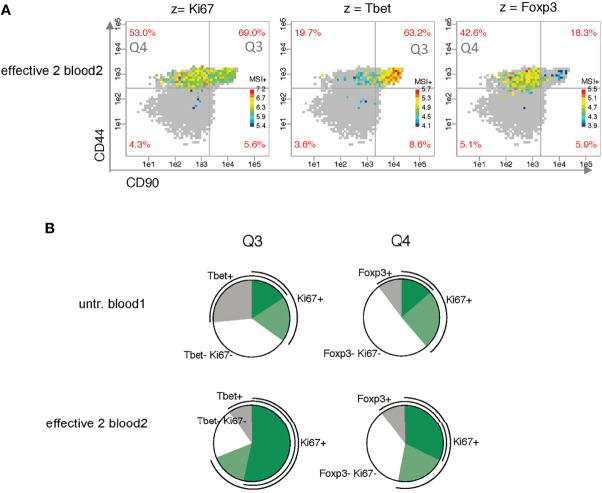
Analysis of the co-expression of the proliferation marker Ki67 and the master transcription factors Tbet and Foxp3. **(A)** Analysis of bin patterns for mean signal intensity of z^+^ cells (MSI^+^) using one exemplary sample for effective therapy (effective 2 blood2) for Ki67, Tbet and Foxp3 using dynamic ranges for each sample. **(B)** Pie charts show differences in Ki67 co-expression between the two exemplary samples (untreated blood1 and effective 2 blood2).

Next, we analyzed differences in expression level between bins taking only z^+^ cells into account (**Figure 5A**). Bins with less than five z^+^ cells are shown in gray. A distinctive pattern is apparent for the two transcription factors (TF) Tbet and Foxp3. The highest and lowest intensities are clearly divided between quadrants Q3 and Q4 and in opposite ways. Ki67 expression, on the contrary, shows no clear tendency to higher level in either of the two upper quadrants.

To investigate the co-expression of Ki67 with the two major TF, we compared the bins for two z parameters per cell in the two exemplary samples. The pie charts clearly show that effective therapy mainly increases the Ki67/TF co-expression sectors of Ki67^+^Tbet^+^ in Q3 and Ki67^+^Foxp3^+^ in Q4 (**Figure 5B**).

## Discussion

A bottleneck in the broader application of cytometric technologies is unresolved data analysis issues. High-dimensional cytometric measurements provide an enormous amount and complexity of quantitative single-cell protein data for millions of cells generated within minutes. Many recent review articles emphasize the need for new approaches and tools in cytometry (2, 9–11, 24). Most of the current analysis strategies are based on clustering and the respective frequencies of the clusters. The main problem with these methods is that they tend to find mainly cell population clusters which are significantly high in phenotypical contrast. If the cell subpopulations are very similar or even partially overlapping, as in the present investigation, then a cluster approach might fail to discriminate these subpopulations (3, 25).

To circumvent this problem, we developed the PRI strategy (13) which is not based on clustering of cells but on grouping of cells according to their expression intensities of two protein parameters (x and y). In a semi-continuous way, the x-y plane is divided into areas of equal size, which can be considered as many mini-gates. All statistical parameters, e.g. mean signal intensity (MSI) for a third parameter (z), can then be plotted per bin in color-coded manner. The approach is useful, for example, when a correlation of the intensity of the z marker with an x or y marker is revealed, or adjacent areas of a z marker have high expression levels and others have low expression levels (Tbet^+^ cells in **Figure 5A**).

PRI is an unsupervised analysis that engineers reproducible bin features and produces bin plots that are intuitively understandable. Other attributes of the PRI approach are summarized in **Table 1**. A major drawback of the PRI approach is that it only works properly with many cells per sample and appropriate markers to obtain enrichment of interesting (sometimes rare) cell subsets in enough bins for reliable statistics.

**Table 1:** I Advantages of PRI.

Characteristics	power of PRI	Comments
unsupervised	++	
reproducible results	++	
quantitative analysis (single cell resolution)	++	PRI: retained for analysis
intuitive interpretation	++	
simple statistical analysis (robust)	++	
sample comparison	++	
integration of experience and expertise	++	
no clustering-based method	++	
no-downsampling	++	downsampling is a disadvantage of many other methods
automatable	+	
low computing power	+	
rapid visualisation	+	
stand-alone application	+	PRI: after pre-processing of cytometry data (e.g. FlowJo)

+ high power of PRI; ++ very high power of PRI.

Our study has produced two main results: it has demonstrated the feasibility of PRI analysis and visualization for high-dimensional mass cytometry data, and it has provided new immunological insights by re-analyzing a public mass cytometry dataset. We revealed two bin plot patterns (CD90/CD44/CD86 and CD90/CD44/CD27 in **Figure 3A**) and five maximum bin plot values (z being each of CD86, CD27, Foxp3, KLRG1 and PDL1 in **Figures 3B**, **4B**) for threshold-independent classification of mice concerning the prognostic of ineffective and effective tumor treatment. Traditionally, frequencies are an important issue in cytometric analysis. Therefore, we manually introduced thresholds to discriminate producing (+) vs. nonproducing cells (-) or for nonproducing and low producing (-/low) vs. high producing (high) cells. Thereby, we discovered seven frequency features (CD86, CD27, Ki67, PDL1, Foxp3 and KLRG1) and one density feature (CD90^high^CD44^-^) showing significant differences between the two classes.

Using our PRI approach, we could visualize in a pseudo-multiparametric manner more specificities of the described Th1 cluster mixture (CD44^+^ CD69^-^ CD62L^-^ CD27^low^ CD90^+^ Tbet^+^) (14). We could further constrain it to CD90^high^ CD86^high^ Tbet^high^ cells. In addition, we mapped with PRI cell subsets regarding co-expression of the proliferation marker Ki67 with Tbet (**Figure 5B**) conforming the expansion of Tbet^+^ subpopulation in parallel to a Foxp3^+^ subpopulation with effective therapy.

There are four major limitations of this study. First, the fact that there are only a limited number of mice per condition in the dataset (3 in each treatment group and 6 in each classification group). Second, the selection of the x and y markers is not unbiased so far. However, the selection was supported by the NRS-score, manual inspection of different combinations and by the identified CD4+ subpopulation of the original article. Third, we cannot rule out that there are other interesting subpopulations in the CD4+ dataset which are not represented by CD90-CD44 plane. Fourth, in principle, there is a risk of discovering patterns by chance even in random data if enough plots are examined. To address this issue, future studies could include markers with no effect as a null model.

After demonstrating the general power of PRI analysis and visualization for high-dimensional mass cytometry data in this work, we will take advantage of PRI to develop an automated workflow and incorporate a machine learning approach.

## Data Availability Statement

Publicly available datasets were analyzed in this study. This data can be found here: https://community.cytobank.org/cytobank/projects/1025, https://github.com/InesHo/PRI-demonstration.

## Author Contributions

RB conceptualized the project. The methodology was drafted by IK, ARy, MS, and YH. SG, RB and YH analyzed the data. RB and IH wrote the original draft for the manuscript. The manuscript draft was reviewed and edited by YH, IH, SG, and RB. The funding for this project was acquired by RB. SB preprocessed the data and provided domain expertise on manual flow and mass flow cytometry analysis. DG, DW, AR, and RB supervised the project. JV provided bioinformatics domain expertise. All authors contributed to the article and approved the submitted version.

## Funding

This work is funded by the German Federal Ministry of Education and Research (BMBF) as part of the e:Med MelAutim project (grant 01ZX1905C to JV and RB).

## Conflict of Interest

The authors declare that the research was conducted in the absence of any commercial or financial relationships that could be construed as a potential conflict of interest.

## Publisher’s Note

All claims expressed in this article are solely those of the authors and do not necessarily represent those of their affiliated organizations, or those of the publisher, the editors and the reviewers. Any product that may be evaluated in this article, or claim that may be made by its manufacturer, is not guaranteed or endorsed by the publisher.
