# Supplementary material for: PRI: Re-Analysis of a Public Mass Cytometry Dataset Reveals Patterns of Effective Tumor Treatments
Source: Front Immunol. 2022 May 3;13:849329. doi: 10.3389/fimmu.2022.849329 (PMC9110672; doi:10.3389/fimmu.2022.849329)
Supplement: Supplementary file 1 [file DataSheet_1.docx]

Supplementary Material

# Supplementary Figures and Tables

## Supplementary Figures

z = CD86

blood 3

blood 2

blood 1

*****

untr. tCD90

ineff. tCD90

(anti-PD-1)

effective 1

(IFN-γ + anti-CD40 +

CD1-allo-IgG)

*****

effective 2

(IFN-γ + anti-CD40 +

B6--allo-IgG)

CD44

CD90

**Supplementary Figure 1.** Dynamic MSI bin plots for CD86 as z parameter with CD90 and CD44 on the x and y axis for all blood samples. In these plots, the z parameter of each sample is color-coded relatively to its own individual minimum-maximum range of MSI. Thresholds were defined by manual inspection for x and y to discriminate CD44^-^ and CD44^+^ cells, as well as CD90^-^/low and CD90high cells. The two exemplary samples (untr. blood1 and effective2 blood2 (IFN-γ + anti-CD40 + B6-allo IgG)) for Fig. 3A and Fig. 4A are marked with an asterisk.

z = CD27

blood 3

blood 2

blood 1

*****

untr. tCD90

ineff. tCD90

(anti-PD-1)

effective 1

(IFN-γ + anti-CD40 +

CD1-allo-IgG)

*****

effective 2

(IFN-γ + anti-CD40 +

B6--allo-IgG)

CD44

CD90

**Supplementary Figure 2.** Dynamic MSI bin plots for CD27 as z parameter with CD90 and CD44 on the x and y axis for all blood samples. In these plots, the z parameter of each sample is color-coded relatively to its own individual minimum-maximum range of MSI. Thresholds were defined by manual inspection for x and y to discriminate CD44^-^ and CD44^+^ cells, as well as CD90^-^/low and CD90high cells. The two exemplary samples (untr. blood1 and effective2 blood2 (IFN-γ + anti-CD40 + B6-allo IgG)) for Fig. 3A and Fig. 4A are marked with an asterisk.

**(A)**

**(B)**

**Supplementary Figure 3.** Descriptive plots for all blood samples. The color indicates the assignment to the treatments. Samples of the effective treatment are shown in shades of green and samples of the ineffective treatment are shown in red and orange. The basis for the calculations is the median of the asinh-transformed signal intensities of all 41 markers across all cells. Euclidian distances between samples’ marker medians were applied in the MDS (A), as well as in the dendrogram using Ward’s hierarchical clustering method (B).

## Supplementary Tables

**Supplementary Table 1**. Panel overview of cell markers of Spitzer et al. Markers with an asterisk are the subset of T-cell markers (22) selected for PRI and PCA analysis. Their associated mean non-redundancy score (NRS) is shown in descending order and from left to right.

| marker | mean NRS | marker | mean NRS | marker | mean NRS | marker | mean NRS |
| --- | --- | --- | --- | --- | --- | --- | --- |
| CD44* | 9,19 | RORgt* | 1,55 | Ter119 | - | cKit | - |
| CD90* | 5,61 | CD62L-FITC* | 1,22 | Ly6G | - | B220 | - |
| CD86* | 4,92 | SiglecF* | 1,19 | IgD | - | NK1.1 | - |
| KLRG1* | 3,47 | CD8* | 1,10 | Pr CD16/32 | - | F4/80 | - |
| Ki67* | 3,33 | PD-L1* | 1,06 | CD49b | - | CD115 | - |
| CD64* | 3,00 | FcER1a* | 1,00 | CD11c | - | CD19 | - |
| T-bet* | 2,93 | CD27* | 0,80 | PyMT | - | IgM | - |
| CD69* | 2,69 | CD45* | 0,65 | CD103 | - | MHC | - |
| Foxp3* | 2,66 | PD-1* | 0,44 | PDCA-1 | - |  |  |
| CD138* | 1,71 | CD3* | 0,34 | Ly6C | - |  |  |
| TCRgd* | 1,71 | CD4* | 0,34 | CD11b | - |  |  |
